# Reintubation following planned extubation: incidence, mortality and risk factors

**DOI:** 10.1186/2197-425X-3-S1-A684

**Published:** 2015-10-01

**Authors:** D Whitmore, T Mahambray

**Affiliations:** St. Helens and Knowsley Teaching Hospitals NHS Trust, Critical Care, Merseyside, United Kingdom

## Introduction

Studies indicate reintubation rates following planned extubation to be between 10-20% in the general ICU population. There is evidence that extubation failure and reintubation can worsen outcome, with studies suggesting ICU mortality rates of between 25-50% in these patients.

## Objectives

To establish how our failed extubation rate and subsequent mortality compare to previously published studies, whilst assessing the impact of previously proposed risk factors for extubation failure.

## Methods

In this retrospective case note study we looked at the outcome of each extubation between 1st October 2013 and 30th September 2014. We included all patients >18 years of age invasively ventilated for >48 hours. We excluded patients proceeding directly to tracheostomy or extubated for withdrawal of care. Failed extubation was defined as the need for reintubation within 5 days of planned extubation. Data was analysed in two groups; ´success´ and ´failure´ of extubation.

## Results

180 patients were initially included in the study, with 105 remaining once exclusion criteria had been applied. There were a total of 141 extubations with a failure rate of 38%. Those in the ´failure´ group had a median length of ICU stay of 14.9 days vs. 8.2 days in the ´success´ group. The ICU mortality in these groups being 10% vs 8% respectively.

In relation to risk factors for extubation failure, there were no clinically significant differences between the ´failure´ and ´success´ groups in terms of: age (58 vs 57 yrs); duration of IPPV (3.3 vs 4 days); fluid balance (+ve 2047 vs 2386 mls.); RSBI (50 vs 36) and APACHE II scores (15 vs 17) respectively. However, 90% of patients with a RSBI >100 immediately prior to extubation did subsequently require reintubation.

Emergency non-invasive ventilation was commenced in 10% of patients post extubation, with 70% subsequently requiring reintubation. The reasons for extubation failure were difficult to determine, but upper airway obstruction was documented as a contributing factor in 19% of failed extubations.

## Conclusions

Reintubation post failed extubation is associated with a prolonged ICU stay, but in our patient population had no impact on ICU mortality. Extubation failure is difficult to predict although a RSBI >100 at the time of extubation appears to place patients at particularly high risk. The use of emergency NIV post extubation has a high failure rate and should not delay reintubation when necessary. Upper airway obstruction still appears to be an important cause of extubation failure and we would recommend a ´cuff leak´ test prior to extubation.
